# Heatline visualization of MHD natural convection heat transfer of nanofluid in a prismatic enclosure

**DOI:** 10.1038/s41598-021-89814-z

**Published:** 2021-05-26

**Authors:** Tarikul Islam, Md. Nur Alam, Muhammad Imran Asjad, Nazma Parveen, Yu-Ming Chu

**Affiliations:** 1 Department of Mathematics, Bangabandhu Sheikh Mujibur Rahman Science and Technology University, Gopalganj, 8100 Bangladesh; 2 Department of Mathematics, Pabna University of Science and Technology, Pabna, Bangladesh; 3 Department of Mathematics, University of Management and Technology, Lahore, Pakistan; 4 Department of Mathematics, Bangladesh University of Engineering & Technology, Dhaka, Bangladesh; 5 Department of Mathematics, Huzhou University, Huzhou, 313000 P. R. China

**Keywords:** Applied mathematics, Mechanical engineering

## Abstract

Temperature transfer by virtue of natural convection for visualizing heat transport characteristics through heatline method within a prismatic cavity filled with Cu-H_2_O nanofluid considering two different temperature boundary conditions is performed numerically. Two top inclined walls are warmed-up at low temperature whilst the bottom wall is heated two different heated conditions such as uniform temperature condition and linear temperature condition. Two vertical walls are insulated. Finite element technique of Galerkin weighted residual form is employed for solving nonlinear partial differential equations for numerical calculation. Heatlines, isotherm contours, streamline contours, and Nusselt number are employed for displaying numerical simulated results for the model parameters entitled nanoparticles volume fraction, Hartmann number and Rayleigh number. The outcomes indicate that heat transfer rate has a significant impact on thermal boundary condition and shape of the nanoparticles. The temperature transfer value enhances significantly for higher Rayleigh number as well as nanoparticles volume fraction. Hartmann number has a positive impact on fluid flow and temperature transport. The characteristics of heat transport using heatlines method are also performed for predicting the better energy transform compared to isotherm contours. In addition, different types of nanofluids are also employed to examine the best heat transport performance.

## Introduction

Nanofluids consist nanoparticles such as Co, Zn, Ag, Cu, Al_2_O_3_, TiO_2_, and Fe_3_O_4_ etc. and conventional fluid named water, engine oil, and kerosene etc. These nanofluids are widely used as noble fluid in many engineering and industrial applications. The main objectives of the investigation of nanofluids is that finding of height feasible solutions using low cost. In last few years, the interdisciplinary investigations of nanofluids are increased for the wonderful real life engagement in industries and engineering. For instance, solar water heating, heat exchangers, fuel cell, diesel combustion, vehicle’s engines, nuclear systems, domestic refrigerator, and lubrications. Choi^1^ investigated about the technology of nanofluids for its present and future enrollment. Buongiorno^2^ performed convective temperature transport in nanofluids. Jou et al.^3^ investigated numerically about free conventional temperature augmentation within a rectangle shape cavity using nanofluids. Ece et al.^4^ performed about magnetic field influence on free convective flow using warm-up and cold adjoining walls within a rectangle shape enclosure. Das et al.^5^ investigated nonofluids and its application in science and technology. Oztop et al.^6^ studied about convective flow within partly warmed-up rectangle shape cavity using nanofluids.

Temperature transference by buoyancy introduced flowing becomes a monumental engineering wonderment now-a-days. Natural convection heat transfer and cooling systems in closed enclosures are very significant wonderment in geophysical, geothermal reservoirs, heat exchanger design, mechanical and electronic industries. Many scientists and researchers are highly motivated to investigate the distribution of temperature transport, heat flow and flow pattern. For instance, Ghasemi et al.^7^ performed regarding free convective temperature flow using CuO-water nanofluids within the dangling cavity. Rahmam et al.^8^ investigated about combined convective flow within a rectangle shape enclosure. Wong et al.^9^ investigated about present as well as future enrolments of nanofluids. Seleh et al.^10^ investigated about free convectional temperature flow employing nanofluids within trapezoid shape cavity. Arani et al.^11^ investigated as regards natural convectional flow into nanofluids within a square enclosure using warmed blade. Basak et al.^12^ investigated regarding the analysis of heatlines upon free convectional flow employing nanofluids within a square enclosure for several warmed-up boundary systems. Cheikh et al.^13^ investigated regarding fee convectional flow of nanofluids within a square cavity employing a non-uniform warmed-up conditions. Free convectional temperature flow into nanofluids using constant heat flux was researched by Seyyedi et al.^14^. Salma et al.^15^ investigated about free convectional flow of nanofluids within a prismatic enclosure.

In two dimensional convection heat transfer process, heatline is the best method of understanding and analyzing the heat flow. The real path of convection heat transfer or the heat recovery system is visualized by heatline method. The streamlines completely illustrate the flow of the fluid whereas isotherm contours narrate mere the distributions of temperature which are not adequate for visualizing of temperature transportation. The heatline method is similar with streamlines that is important for analyzing fluid motion. Heatline techniques is the best measures for the finding of temperature recovery systems as well as actual temperature transfer way by the virtue of convection. Heatlines typically illustrate temperature functions that satisfy energy equation whilst stream function satisfy conservation of mass equations. Heatline is associated with Nusselt number that depends on non-dimensional form of conversion. The heat flux lines are represented by heatlines which illustrate the trajectory of heat flow that are normal to the isothermal lines in convection temperature transport. The heatlines are represented mathematically by heat function and each heat function corresponds a constant function. Kimura and Bejan^16^ was first introduced the concept of heatline. Salma et al.^17^ further studied about heatlines visualization on magneto-hydrodynamics combined convective flow including a heated block. Uddin et al.^18^ researched about the fundamentals concepts as well as the applications of nanofluids. Alam et al.^19^ investigated regarding the heatlines exploration upon free convective flowing as well as temperature transfer within prismatic cavity employing FEM. Alsabery et al.^20^ investigated about the experiment of heatlines analysis over convectional flow within a square enclosure using sinusoidally temperature mutations. Sheikha et al.^21^ investigated about convective temperature flow of nanofluids within a trapezoidal cavity.

The influence of magnetohydrodynamic (MHD) on natural convection flow as well as heat transfer of nanofluids have been received operative attention in current years on account of their extensive diversity applications in the area of science, industry and engineering. For this reasons, many scientists and researchers have been motivated to perform numerical simulation for examining the fluid flow, heat transport and temperature flow. Kalbani et al.^22^ researched about convective temperature flow into nanofluids within a square cavity for the existence of magneto-hydrodynamics. Latifa et al.^23^ investigated about fee convectional temperature flow within a square cavity employing magneto-hydrodynamics. Sheremet et al.^24,25^ studied natural convection of nanofluid in different cavities using Buongiorno’s mathematical model. They found that additional nanoparticles intensify convection flow. Different study^26–28^ also investigated about the natural convection of nanofluids inside cavities. The main intention of this investigation is to analyze the convectional flow within a prismatic cavity utilizing copper–water nanofluid for visualizing the temperature flow as well as finding a proficiency path of temperature changing. The impacts of nanoparticles volume fraction, Hartmann number and Rayleigh number upon temperature allocation as well as fluid flow within a prismatic cavity are performed numerically and observed them physical point of spectacle.

## Problem formulation

### Physical model

**Figure 1:**
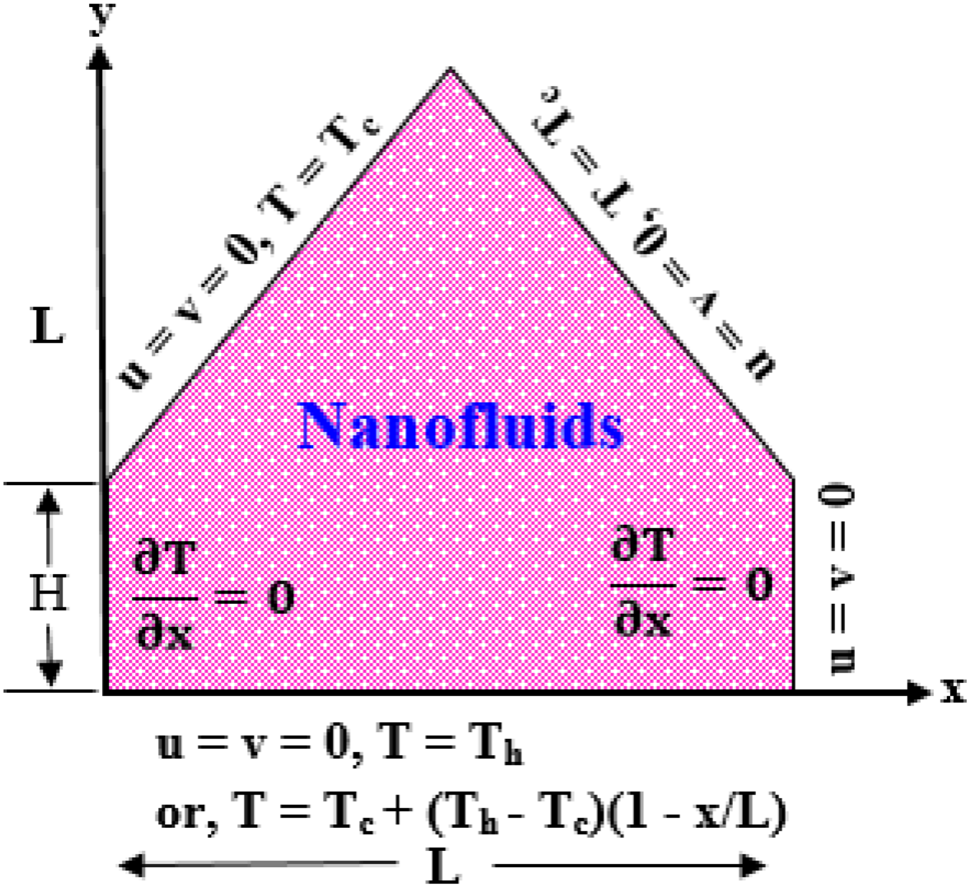
Schematic view of prismatic enclosure.

The physical model taking into account a two-dimension, laminar, and incompressible free convection flow within prism shape cavity charged uniformly by Cu-H_2_O nanofluid. We have taken water (H_2_O) as base fluid and copper (Cu) as nanoparticles. $L$ represents height and base wall length of the enclosure. A natural convection has been introduced by the difference of the temperature between heated and cold walls. The bottom wall is warmed-up at $T = T_{c} + (T_{h} - T_{c} )(1 - x\,/L)$ (linearly) and $T = T_{h}$ (uniformly) whilst the top inclined walls are warmed-up at low heat $T = T_{c}$. The vertical walls are kept at insulation. The bottom wall represents $x -$ coordinate and left vertical wall represents $y -$ coordinate. The gravitational acceleration acts to the negative y-direction. It is considered that the nanoparticles are dispersed homogenously into the base fluid and no dynamical and thermal slip happens between base fluid and nanoparticles. No slip walls are considered for all solid boundaries. Different types of base fluid such as kerosene and Ethylene Glycol (EG) and different types of nanoparticles such as cobalt (Co), alumina (Al_2_O_3_) and titanium oxides (TiO_2_) are used to investigate average Nusselt number at heated wall.Fig. 1 showed a schematic spectacle of geometry attached to the Coordinate system and thermophysical properties of nanoparticles including base fluids are presented in Table 1.

**Table 1:** Thermo-physical characteristics rigid particles and the base fluid (see^33^).

Base Fluid/Nanoparticles	c_p_ [JKg^−1^ K^−1^]	ρ [km^−3^]	k [Wm^−1^ K^−1^]	μ [Kgm^−1^ s^−1^]	β × 10^–5^ [K^−1^]	σ [Sm^−1^]	Pr
Water (H_2_O)	4179	997.1	0.613	0.001003	21	5.5 × 10^–6^	6.8377
Kerosene	2090	780	0.149	0.00164	99	6.0 × 10^–10^	23.004
Ethylene Glycol (EG)	2382.1	1117.48	0.2492	0.022	57	1.07 × 10^–8^	210.3
Cu	385	8933	400	–	1.67	5.96 × 10^7^	–
Co	420	8900	100	–	1.3	1.602 × 10^7^	–
Al_2_O_3_	765	3970	40	–	0.85	3.5 × 10^7^	–
TiO_2_	686.2	4250	8.9538	–	0.90	2.6 × 10^7^	–

### Mathematical model

The governing equations of aforementioned assumptions in Cartesian coordinates are:

1 $$ \frac{\partial u}{{\partial x}} + \frac{\partial v}{{\partial y}} = 0 $$

2 $$ \rho_{nf} \left( {u\frac{\partial u}{{\partial x}} + v\frac{\partial u}{{\partial y}}} \right) = - \frac{\partial p}{{\partial x}} + \mu_{nf} \left( {\frac{{\partial^{2} u}}{{\partial^{2} x}} + \frac{{\partial^{2} u}}{{\partial^{2} y}}} \right) $$

3 $$ \rho_{nf} \left( {u\frac{\partial v}{{\partial x}} + v\frac{\partial v}{{\partial y}}} \right) = - \frac{\partial p}{{\partial y}} + \mu_{nf} \left( {\frac{{\partial^{2} v}}{{\partial^{2} x}} + \frac{{\partial^{2} v}}{{\partial^{2} y}}} \right) + \left( {\rho \beta } \right)_{nf} g(T - T_{c} ) - \sigma_{nf} B_{0}^{2} \,v $$

4 $$ u\frac{\partial T}{{\partial x}} + v\frac{\partial T}{{\partial y}} = \alpha_{nf} \left( {\frac{{\partial^{2} T}}{{\partial^{2} x}} + \frac{{\partial^{2} T}}{{\partial^{2} y}}} \right) $$

### Boundary conditions

On the bottom wall:

5a $$ {\text{case I:}}\;u = 0,v = 0,T = T_{h} $$

5b $$ {\text{case II:}}\;u = v = 0,T = T_{c} + (T_{h} - T_{c} )\left( {1 - \frac{x}{L}} \right) $$

5c $$ {\text{On}}\;{\text{the}}\;{\text{top}}\;{\text{inclined}}\;{\text{wall:}}\;u = v = 0,T = T_{c} $$

5d $$ {\text{On}}\;{\text{the}}\;{\text{perpendicular}}\;{\text{wall:}}\;u = 0,v = 0,\frac{\partial T}{{\partial x}} = 0 $$

### Physical and thermal properties of nanofluids

For enhancing the thermal performance of nanofluids, the thermal and physical properties of nanofluids are important. The following physical and thermal characteristics of nanofluids are taken into consideration and listed as viscosity, density, thermal diffusivity, heat capacitance, thermal conductivity, thermal expansion coefficient and electrical conductivity, respectively (see Kalbani et al.^29^):

6 $$ \mu_{nf} = \mu_{bf} \frac{1}{{(1 - \phi )^{2.5} }} $$

7 $$ \rho_{nf} = (1 - \phi )\,\rho_{bf} + \phi \,\rho_{sp} $$

8 $$ \alpha_{nf} = \frac{{k_{nf} }}{{\left( {\rho c_{p} } \right)_{nf} }} $$

9 $$ (\rho c_{p} )_{nf} = (1 - \phi )\,(\rho c_{p} )_{bf} + \phi \,(\rho c_{p} )_{sp} $$

10 $$ \frac{{k_{nf} }}{{k_{bf} }} = \frac{{k_{sp} + (n - 1)k_{bf} - (n - 1)(k_{bf} - k_{sp} )\phi }}{{k_{sp} + (n - 1)k_{bf} + (k_{bf} - k_{sp} )\phi }} $$

11 $$ (\rho \beta )_{nf} = (1 - \phi )\,(\rho \beta )_{bf} + \phi \,(\rho \beta )_{sp} $$

12 $$ \sigma_{nf} = \frac{{\sigma_{sp} + 2\sigma_{bf} - 2(\sigma_{bf} - \sigma_{sp} )\phi }}{{\sigma_{sp} + 2\sigma_{bf} + (\sigma_{bf} - \sigma_{sp} )\phi }}\sigma_{bf} $$

## Dimensional analysis

We introduce the following non-dimensional variables for reducing the Eqs. (1)–(4) into non-dimensional form including boundary conditions (5a)–(5d):

13 $$ X = \frac{x}{L},\,Y = \frac{y}{L},U = \frac{uL}{{\alpha_{bf} }},\,V = \frac{vL}{{\alpha_{bf} }},\,\theta = \frac{{T - T_{c} }}{{T_{h} - T_{c} }},P = \frac{{pL^{2} }}{{\rho_{nf} \alpha_{bf}^{2} }} $$

The governing equations in dimensionless form are expressed as:

14 $$ \frac{\partial U}{{\partial X}} + \frac{\partial U}{{\partial Y}} = 0 $$

15 $$ U\frac{\partial U}{{\partial X}} + V\frac{\partial U}{{\partial Y}} = - \frac{{\rho_{bf} }}{{\rho_{nf} }}\frac{\partial P}{{\partial X}} + Pr\left( {\frac{{\rho_{bf} }}{{\rho_{nf} }}} \right)\left( {\frac{{\partial^{2} U}}{{\partial^{2} X}} + \frac{{\partial^{2} U}}{{\partial^{2} Y}}} \right) $$

16 $$ U\frac{\partial V}{{\partial X}} + V\frac{\partial V}{{\partial Y}} = - \frac{{\rho_{bf} }}{{\rho_{nf} }}\frac{\partial P}{{\partial Y}} + Pr\left( {\frac{{\rho_{bf} }}{{\rho_{nf} }}} \right)\left( {\frac{{\partial^{2} V}}{{\partial^{2} X}} + \frac{{\partial^{2} V}}{{\partial^{2} Y}}} \right) + \frac{{\left( {\rho \beta } \right)_{nf} }}{{\rho_{nf} \beta_{bf} }}RaPr\theta - \frac{{\rho_{bf} }}{{\rho_{nf} }}\,\frac{{\sigma_{nf} }}{{\sigma_{bf} }}Ha^{2} \Pr V $$

17 $$ U\frac{\partial \theta }{{\partial X}} + V\frac{\partial \theta }{{\partial Y}} = \left( {\frac{{\alpha_{nf} }}{{\alpha_{bf} }}} \right)\left( {\frac{{\partial^{2} \theta }}{{\partial^{2} X}} + \frac{{\partial^{2} \theta }}{{\partial^{2} Y}}} \right) $$

In the above governing dimensionless equations, $Ra = \frac{{g\beta_{bf} \left( {T_{h} - T_{c} } \right)L^{3} }}{{\upsilon_{bf} \,\alpha_{bf} }}\,\,$ is the Rayleigh number, $\Pr = \frac{{\upsilon_{bf} }}{{\alpha_{bf} }}$ is the Prandtl number and $Ha = B_{o} L\sqrt {\sigma_{bf} /\mu_{bf} }$ is the Hartmann number.

### Non-dimensional forms of the boundary conditions

On the bottom wall:

18a $$ {\text{Case I:}}\;U = 0,V = 0,\theta = 1 $$

18b $$ {\text{Case II:}}\;U = 0,V = 0,\theta = 1 - X $$

18c $$ {\text{On}}\;{\text{the}}\;{\text{top}}\;{\text{inclined}}\;{\text{walls:}}\;U = 0,V = 0,\theta = 0 $$

18d $$ {\text{On}}\;{\text{the}}\;{\text{left}}\;{\text{and}}\;{\text{right}}\;{\text{vertical}}\;{\text{walls:}}\;u = 0,v = 0,\frac{\partial \theta }{{\partial X}} = 0 $$

### Nusselt number calculation

The local and average Nusselt number at the bottom warmed-up wall is expressed respectively as:

19 $$ Nu_{L} = \frac{{Lq_{w} }}{{k_{bf} \left( {T_{h} - T_{c} } \right)}},\;\;{\text{where}}\;q_{w} = - k_{nf} \left( {\frac{\partial T}{{\partial y}}} \right)_{y = 0} $$

20 $$ Nu_{av} = - \left( {\frac{{k_{nf} }}{{k_{bf} }}} \right)\int\limits_{0}^{1} {\frac{\partial \theta }{{\partial Y}}} dX $$

## Computational process

The governing non-dimensional Eqs. (14)–(17) including boundary conditions (18a)–(18d) are performed applying finite element analysis of Galerkin weighted residual form. Zienkiewicz et al.^30^ described the numerical technique elaborately. Firstly, the non-uniform triangular element are formed by discretizing the domain of the solution into limited grid numbers. Six node triangular elements are applied for the progression of this method. Then, integral equations are formed from governing PDEs equations using Galerkin weighted residual technique. Gauss’s quadrature technique is also employed in every part of the integral equations. These equations are also modified using boundary conditions. Newton–Raphson iteration technique is imposed into these algebraic equations for solving these algebraic equations in matrix form. The convergence criterion of this method is imposed as $\left| {{\rm M}^{n + 1} - {\rm M}^{n} } \right| \le 10^{ - 5}$, where ${\rm M}$ represent $U,\,\,V,\,\,\theta$ as dependent variables and $n$ is iteration number.

### Grid independence test

**Figure 2:**
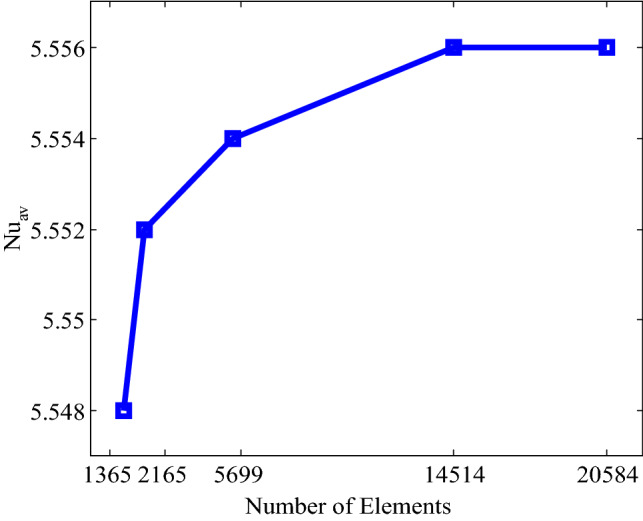
Average Nusselt number of various elements with $Ra = 10^{5}$, $\Pr = 6.8377,$ $n = 3,$ and $\phi = 0.04$.

A comprehensive grid independence experiment is employed to ensure the grid independent solution for the present problem of the prismatic enclosure when $Ra = 10^{5}$, $\phi = 0.04$ and $n = 3$ (spherical shape of nanoparticles). Five different non-uniform grid systems within resolution are employed named normal, fine, finer, extra fine, and extremely fine including elements 1365, 2116, 5699, 14,514, and 20,584. For the above mentioned elements, the average Nusselt number $(Nu_{av} )$ is calculated for understanding the grid fineness that is shown in Fig. 2. The elements 14,514 represents a little difference with the outcomes obtained for the elements 20,584. Hence, elements size 14,514 are employed for numerical calculations to obtain required grid independent solution.

### Code validation

**Figure 3:**
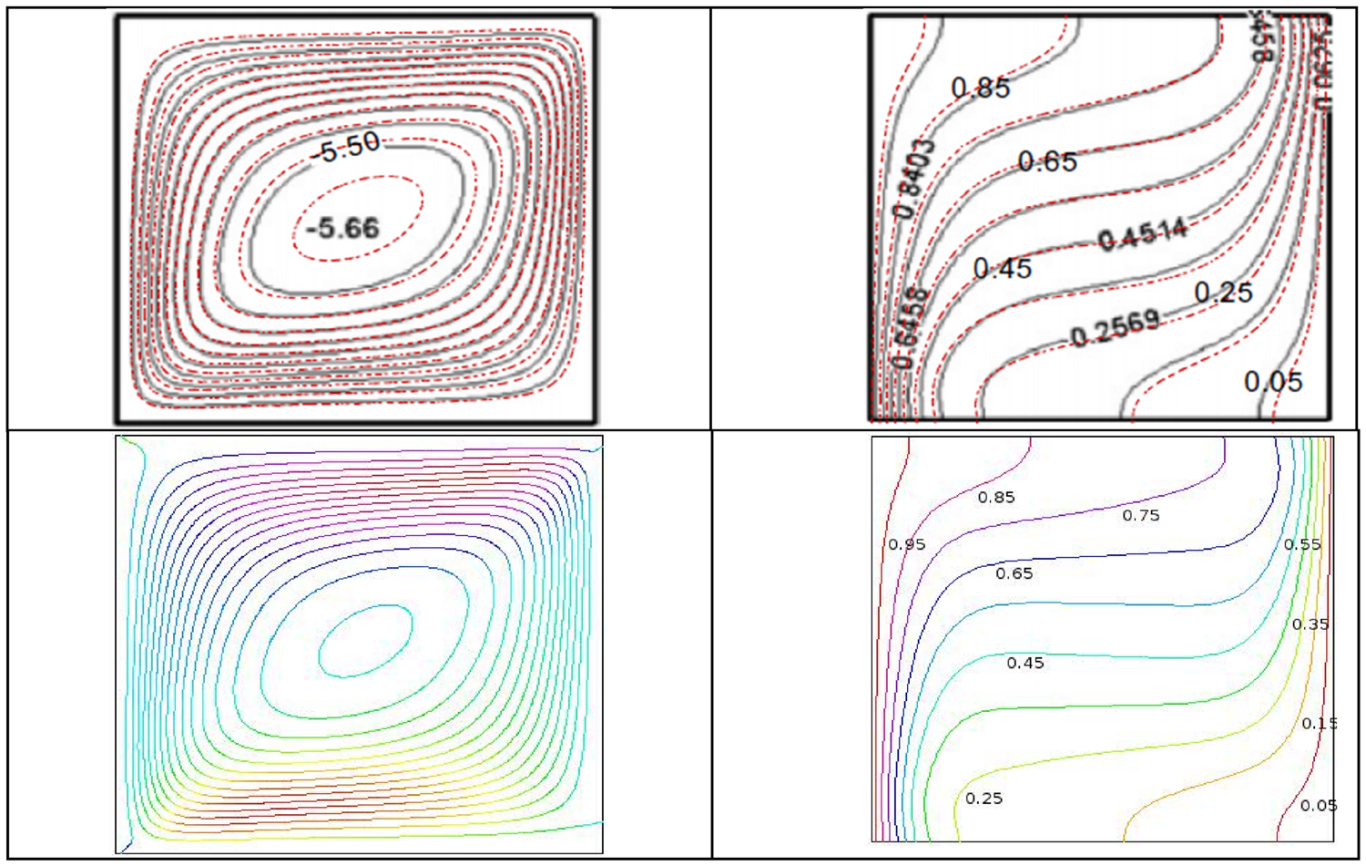
Compare between present outcomes (bottom row) and Ghasemi et al.^31^ (top row) when $$\phi = 0.03,\,$$$$Ra = 10^{5}$$ and $$Ha = 30$$.

The present outcomes are compared with Ghasemi et al.^24^ to check-out the accuracy using streamline contours (left column) and isotherm contours (right column) when $\phi = 0.02,$ $Ra = 10^{5}$ and $Ha = 30$, which is shown in Fig. 3. The present numerical code also validate through numerical data with Ghasemi et al.^31^ for different Rayleigh number and nanoparticles volume fraction considering two dimensional natural convection flow of Al_2_O_3_-water nanofluid under the presence of magnetic effect ($Ha = 30$). The present numerical code also compared with Wan et al.^32^ considering $\Pr = 0.07$ and square cavity filled with air. The current code is transferred for air filled square cavity for comparison. The outcomes represents a good compliance using for employing current numerical code in Table 2.

**Table 2:** Comparison through numerical data of mean Nusselt number $(Nu_{av} )$ with Ghasemi et al. ^31^ and Wan et al. ^32^ for different Rayleigh number and nanoparticles volume fraction.

$\phi = 0$				$\phi = 0.02$	
$Ra$	Ghasemi et al. ^24^	Wan et al. ^25^	Present Study	Ghasemi et al. ^24^	Present Study
10^3^	1.002	1.117	1.002	1.060	1.060
10^4^	1.183	2.254	1.182	1.212	1.208
10^5^	3.150	4.598	3.138	3.138	3.097
10^6^	7.907	8.976	7.820	7.979	7.796

## Result and discussion

The obtained outcomes are presented employing streamline contours, isotherm contours and heatlines for analyzing free convection temperature flow as well as fluid flow within a prismatic enclosure. The spherical shape copper nanoparticles $(\,n = 3)$ are treated into water base fluid. The numerical results have been discussed for various parameters named nanoparticles volume fraction $(\phi )$, Hartmann number $(Ha)$ and Rayleigh number ${\text{(Ra)}}$ upon liquid flow, heat transfer as well as temperature transport characteristics exercising isotherm lines, streamlines, heatlines, local and average Nusselt number using two distinct temperature boundary conditions along horizontal wall.

**Figure 4:**
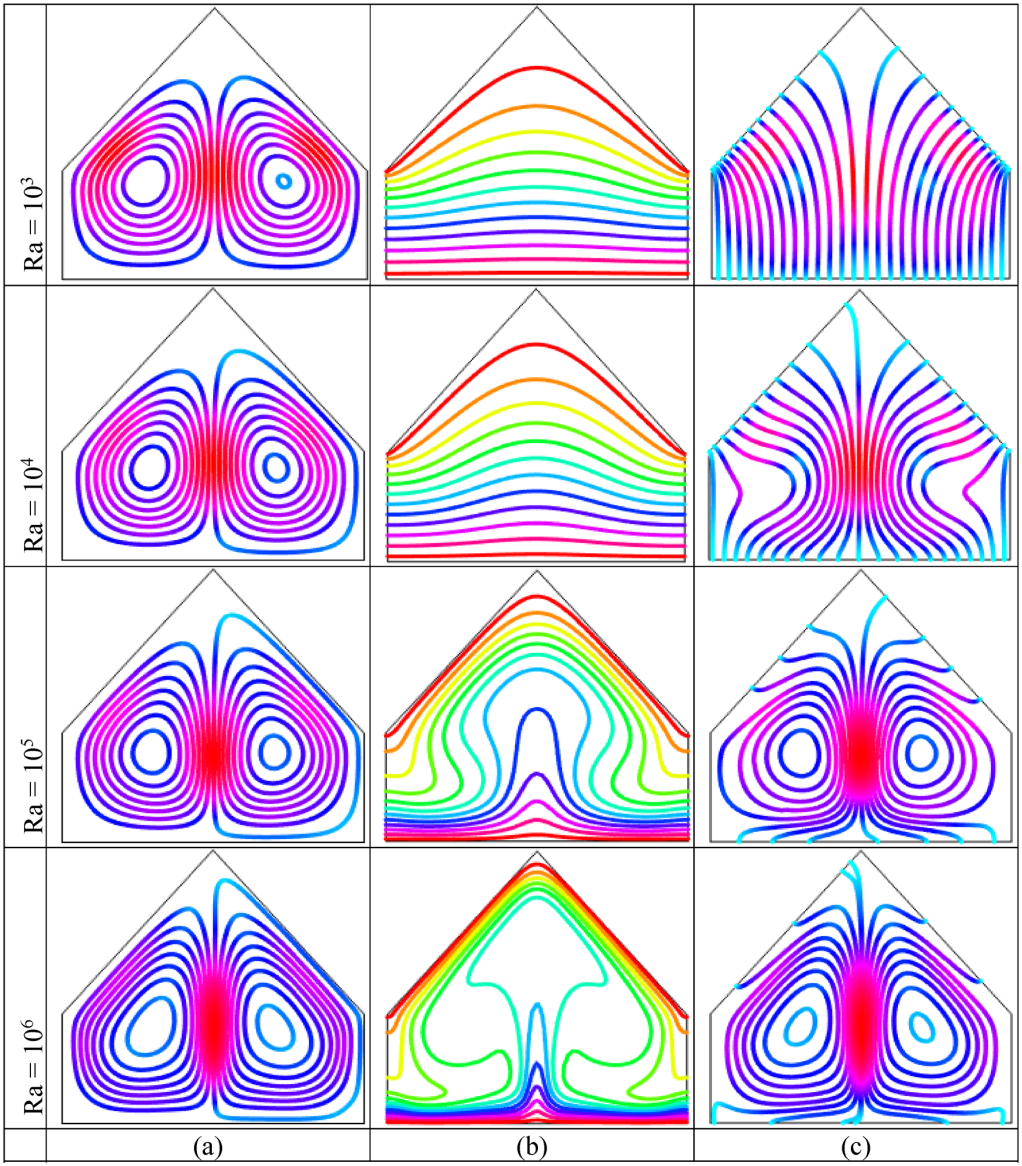
Outcomes of Rayleigh number $Ra$ on (**a**) Streamline contours, (**b**) Isotherm contours (**c**) Heatlines for uniformly thermal boundary condition on bottom wall when $\phi = 0.04$ and $Ha = 10$.

Figure 4 displays the impact of Rayleigh number $$Ra$$$$( = 10^{3} - 10^{6} )$$ when $$\phi = 0.04$$ and $$Ha = 10$$ on the streamlines, isotherm contours as well as heatlines using uniform warmed-up condition on horizontal wall. Figure 4(a) shows that two symmetric counter rotating rolls to the central vertical line are formed within cavity with every $$Ra$$. Also, vortex eyes are situated neighbor the center of half cross-sectional of the cavity though there is uniformly heated bottom. The left-hand chamber circulates anti-clockwise direction whereas the right-hand chamber circulates clockwise direction within the enclosure. The physical meaning behind this that the density of cold liquid near the upper inclined walls is higher compare to warmed-up fluid neighbor the bottom wall within enclosure. The weighty fluid travel below whilst comparatively low frequent liquid turns upwards. Then, the bulky liquid thrust into thermal boundary layer neighbor bottom hot wall and accomplishes rotating pattern. For increases of $$Ra$$, the two recirculation cells grow. This introduces that the warmed-up liquid are accelerated more by virtue of buoyancy effect.

The temperature transfer mode (conduction or convection) as well as the utility of temperature are detected by isotherm contours. Figure 4(b) shows, as low Rayleigh number, $$Ra$$$$( = 10^{3} ,10^{4} )$$, isotherm contours are almost parallel neighbor the bottom hot wall that indicate that convection is weaker within the cavity. Conduction is the principle mode of temperature flow within the enclosure on account of uniform warmed-up condition on bottom wall. The low compactness of the isotherm contours are also observed in the cavity center that represents poorly convective temperature flow. For the increasing values of $$Ra$$, isotherm contours turn out over distorted as well as disappeared in the cavity center manufacturing a particular shape as mushroom. For $$Ra = 10^{5} ,10^{6}$$, the particular pattern of isotherms like mushroom indicates that the temperature energy flows within cavity fluid from bottom warmed-up side by virtue of potential buoyancy effects. Figure 4(c) demonstrates that the heatlines are unadulterated and approximately parallel with perpendicular walls resulting the transformation of temperature as a result of conduction. The heatlines consistency neighbor the warmed-up wall enhance as well as pervert for higher buoyancy driven parameter $$Ra$$. The compactness of the heatlines increases at middle of the enclosure due to the strong convection. Furthermore, for higher $$Ra$$, two symmetric small vortices to the central vertical line are created inside the enclosure, which is consistent with the stream function pattern. Therefore, the convection is dominant form of temperature flow at upper buoyancy driven parameter.

**Figure 5:**
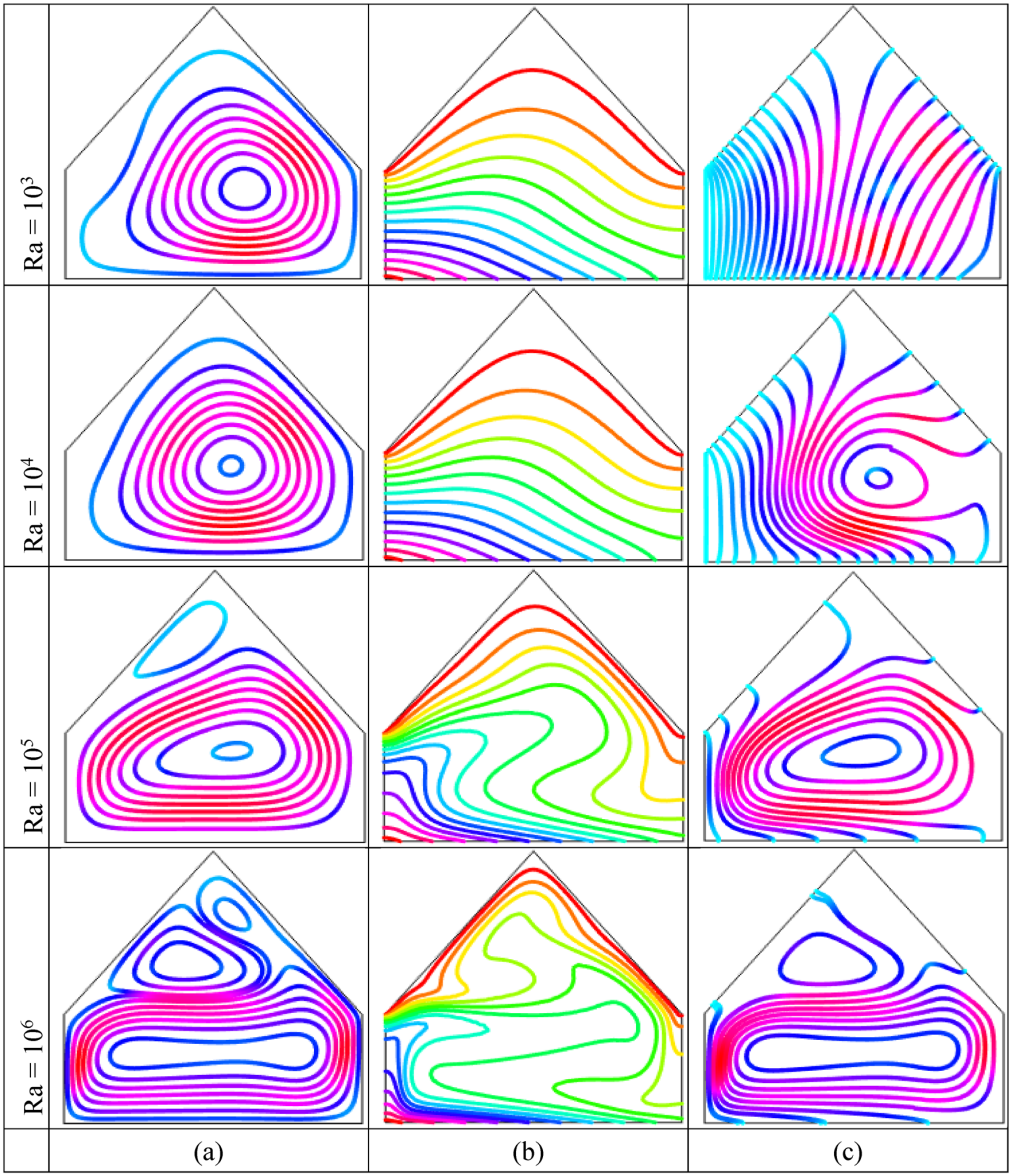
Effect of Rayleigh number $(Ra)$ on (**a**) Streamlines (**b**) Isotherms (**c**) Heatlines for linearly thermal boundary condition on bottom wall when $\phi = 0.04$ and $Ha = 10$.

Rayleigh number $$(Ra = 10^{3} - 10^{6} )$$ effects on the streamline contours, isotherm contours and heatlines when $$\phi = 0.04$$ and $$Ha = 10$$ for linearly warmed condition on bottom wall are displayed in Fig. 5, respectively. Figure 5(a) illustrates that the streamline contours are not comprehensively affected with the particles of the nanofluid within the enclosure. The temperature of horizontal side is upper compared to dangling sides thereby neighboring liquid of bottom wall receives temperature and thereafter these fluid turn out upward to the colder fluid consequently a circulation cell is created consequently temperature transfer value enhances on colder walls. Physical meaning for this is, for the enough temperature differences between warmed up and cooled walls, a potential buoyancy force is constructed within the enclosure. For low $$Ra$$, convection impacts are little conspicuous because of the insignificant performance of inertia force in temperature flow mechanisms. For upper values of $$Ra$$$$( = 10^{5} ,\,10^{6} )$$, streamline contours become decomposed and one potential propagation into the fluid within the cavity is appeared for the increases of the intensity of convective temperature flowing and resulting a secondary circulation is developed on the upper corners neighbor the inclined walls. Figure 5(b) shows, for less $$Ra$$$$( = 10^{3} ,\,10^{4} )$$, parallel isotherm contours neighbor warmed-up wall are observed because of potential conduction form of temperature distributions. For upper Rayleigh number, exceeding distorted isotherm contours are also noticed at the cavity center because of the increases of the convection within the enclosure. For $$Ra = 10^{6}$$, the compactness and high dense of the isotherm pattern is noticed neighbor horizontal warmed-up and dangling roof cold walls that represents high temperature gradient at that regions.

To represents the temperature flow within the enclosure, conductive temperature flux $$\left( { - \frac{\partial \theta }{{\partial X}},\,\frac{\partial \theta }{{\partial Y}}} \right)$$ is employed for heat function whereas convective temperature flux $$\left( {U\theta ,\,V\theta } \right)$$ is employed for displaying temperature flow. Heatlines represent the way of temperature flowing that arises form warmed-up regime whereas finish at cold regime. Figure 5(c) illustrates that the heatlines are distributed almost indiscriminately according clockwise and counterclockwise circulations for less $${\text{Ra}}$$$$( = 10^{3} )$$ from horizontal warmed-up side to upper inclined cold sides due to low strength of fluid flow as well as temperature flowing mainly by conduction.

As increases ${\text{Ra}}$, the flow intensity increases significantly, the pattern of the heatlines are gradually decomposed and an additional tiny rolling chamber is created in upper corner within enclosure. The intense of temperature flow is higher neighbor the bottom warmed wall compared with upper cold wall. Therefore, for the determination of temperature flow as well as the analysis of the temperature transfer, heatlines are sufficient mechanisms. In addition, heatlines appearances as streamline contours for higher ${\text{Ra}}$.

**Figure 6:**
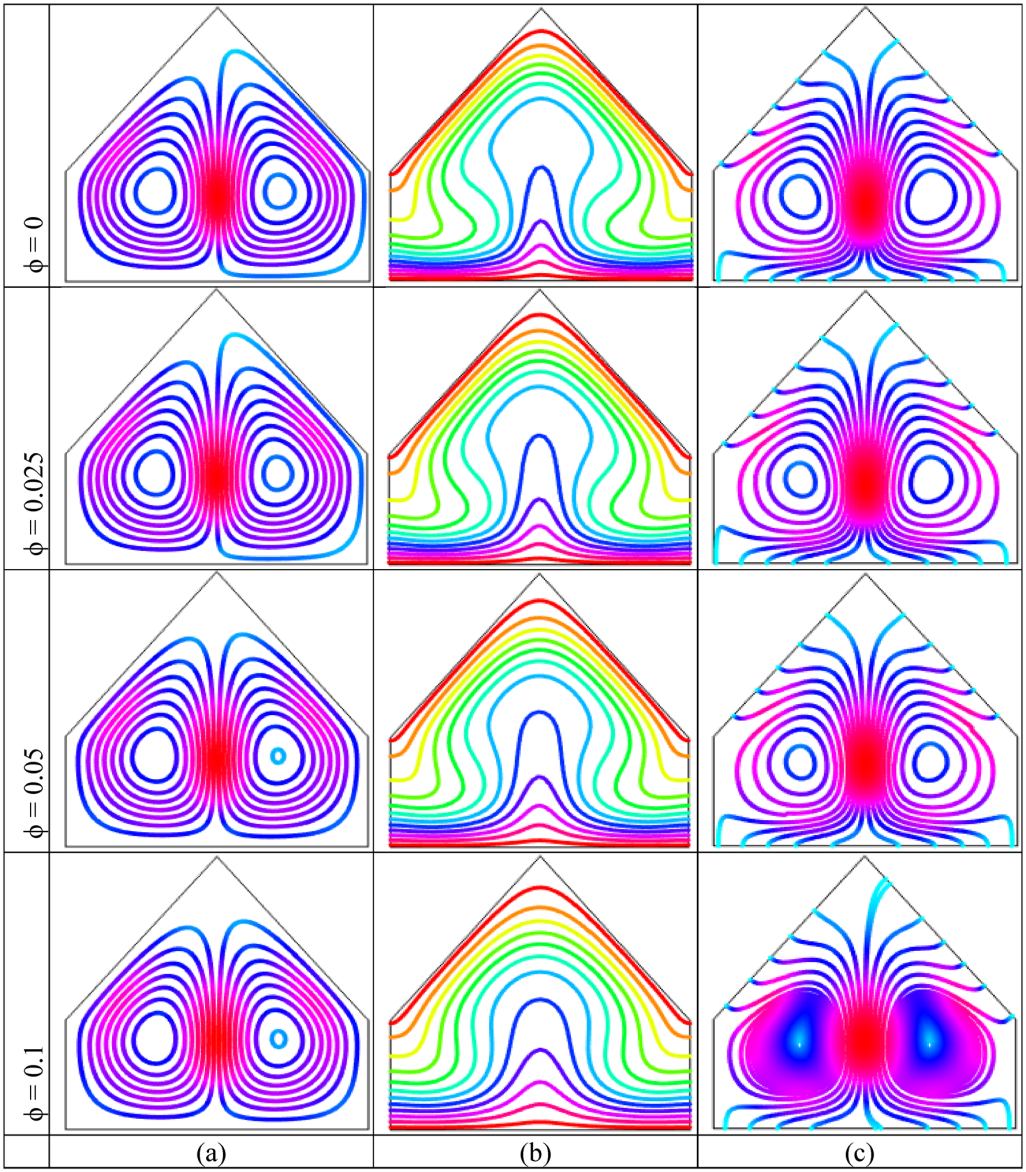
Effect of volume fraction of nanoparticles $(\phi )$ on (**a**) Streamlines (**b**) Isotherms (**c**) Heatlines for uniformly thermal boundary condition on bottom wall when $Ra = 10^{5}$ and $Ha = 10$.

Figure 6, respectively, demonstrates the outcomes of nanoparticles volume fraction $(\phi = 0,\,\,0.025,\,\,0.05,\,0.1)$ when $Ra = 10^{5}$ upon streamline contours, isotherm contours and heatlines for uniform bottom warmed-up wall. Figure 6(a) depicts that two opposite symmetrical counter rotational chambers to central vertical line are formed within the cavity for uniform heated wall. The left circulation is clockwise sense and the other cell is clockwise sense within the enclosure. This particular form be the outcome of uniform warmed-up boundary condition on horizontal side. The physical reason behind this particular form of streamline contours already narrate. For the increases of $\phi$, the pattern of streamlines are nearly similar kind of unadulterated conduction. Furthermore, adding nanoparticles into base fluid within the cavity, a very little effects of nanofluids on convection is observed.

Figure 6(b) illustrates isothermal contours are discomposed at center plane within cavity manufacturing a particular form as mushroom. This depicts that the energy is streaming within liquid from horizontal warmed-up side. This figure also show that the isotherm contours are nearly parallel neighbor warmed-up and cold walls. The fluid neighbor horizontal warmed-up wall accepts temperature and moves upwards into colder fluid. Consequently, temperature flow enhances at cold wall. This figure depict that there is a dense of the isotherm pattern near the warmed-up and cooled walls which indicate the high temperature gradient in that regions. That circumstance stay same for the addition of nanoparticles within the enclosure. Figure 6(c) depicts that the heatlines are identical for all values of nanoparticles and two symmetric small vortices to the central vertical line are created inside the enclosure, which is consistent with the stream function pattern. For $\phi = 0.1$, the compactness of the heatlines increases at middle of the enclosure due to the strong convection.

**Figure 7:**
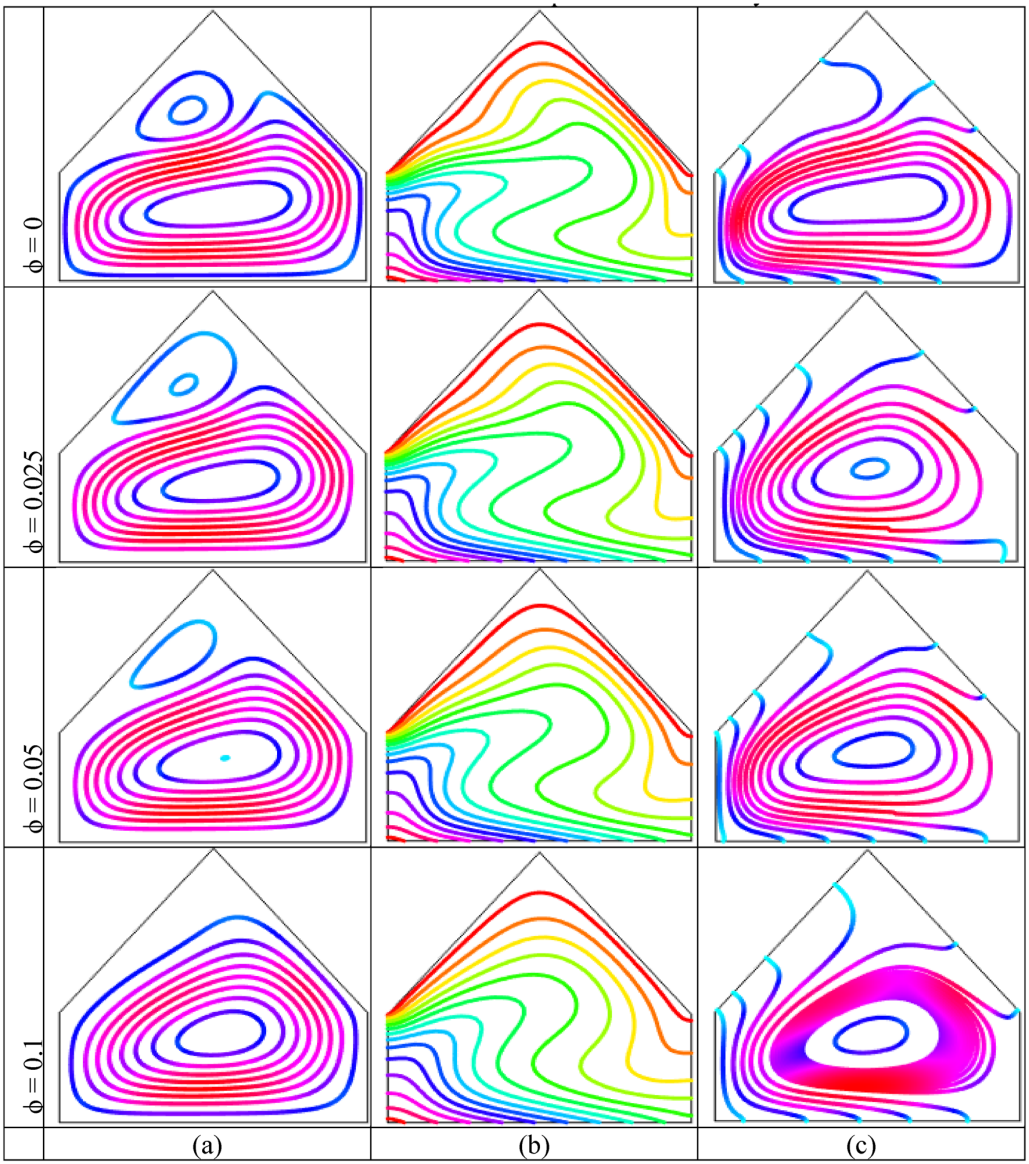
Impact of nanoparticles volume fraction $(\phi )$ on (**a**) Streamlines (**b**) Isotherms (**c**) Heatlines for linearly thermal boundary condition on bottom wall when $Ra = 10^{5}$ and $Ha = 10$.

Figure 7, respectively, illustrate the impact of the nanoparticles volume fraction $(\phi = 0,\,\,0.025,\,\,0.05,\,0.1)$ with fixed $Ra = 10^{5}$ upon the streamline contours, isotherm contours and heatlines for linearly bottom heated wall. Figure 7(a) demonstrate the streamlines are parallel to each other neighbor warmed-up wall because of the conduction as well as a secondary vortex is developed on the upper corners neighbor inclined wall within the cavity. For higher value of nanoparticles the isotherms are distorted and one massive vortex is noticed at the middle of the enclosure. Figure 7(b) narrates that the parallel isotherms neighbor warmed-up and cooled walls. We have already observed this similar particular form. Figure 7(c) shows the heatlines are compact in the middle within the cavity that indicate high temperature flow region. The streamlines are also almost parallel neighbor the bottom warmed-up wall and similar to isotherms what we have already observed. For the increasing values of the nanoparticles the density of the heatlines increase at the center of the enclosure.

**Figure 8:**
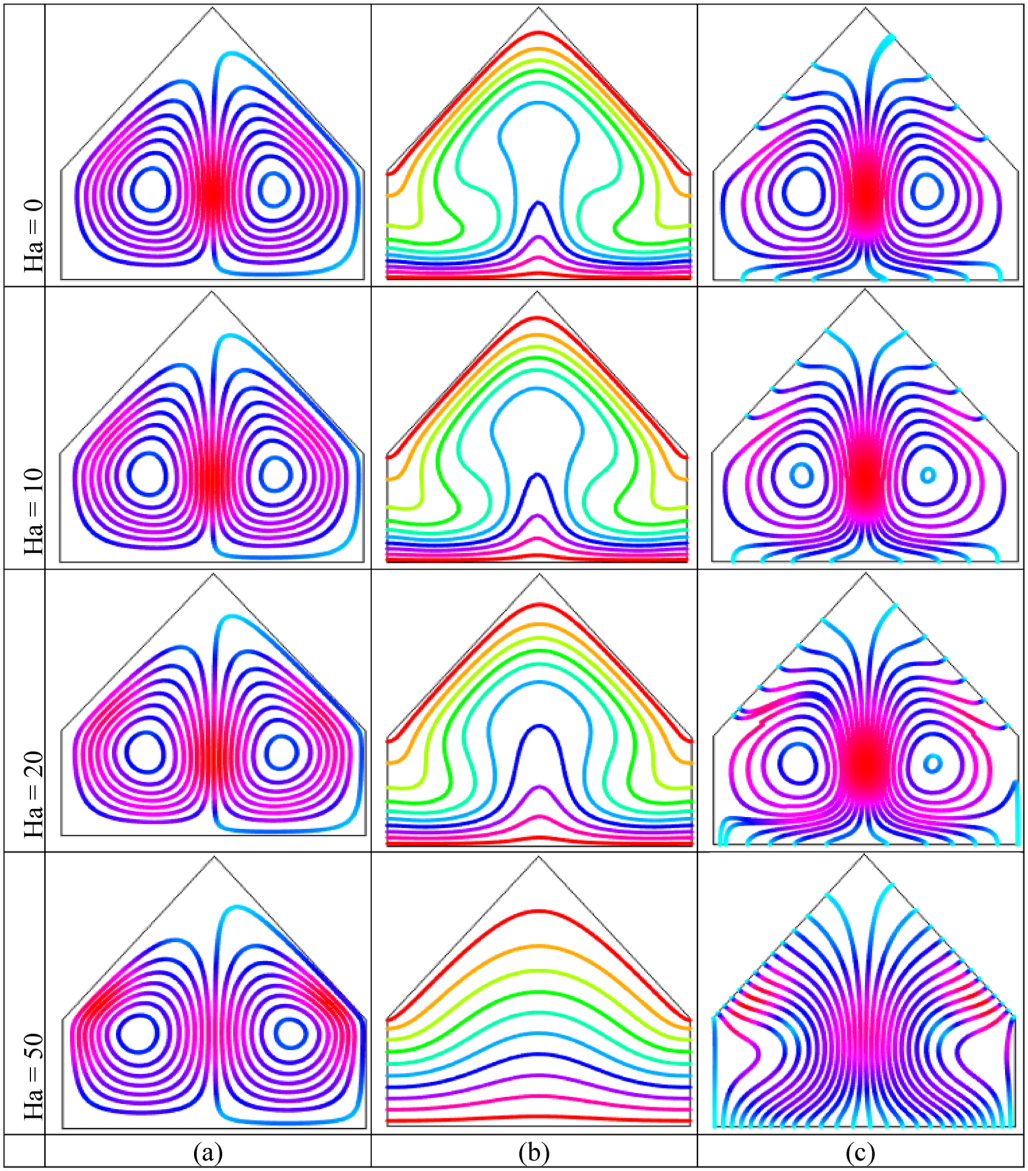
Outcomes of Hartmann number $(Ha)$ on (**a**) Streamline contours, (**b**) Isotherm contours (**c**) Heatlines for uniformly thermal boundary condition on bottom wall when $\phi = 0.04$ and $Ra = 10^{5}$.

**Figure 9:**
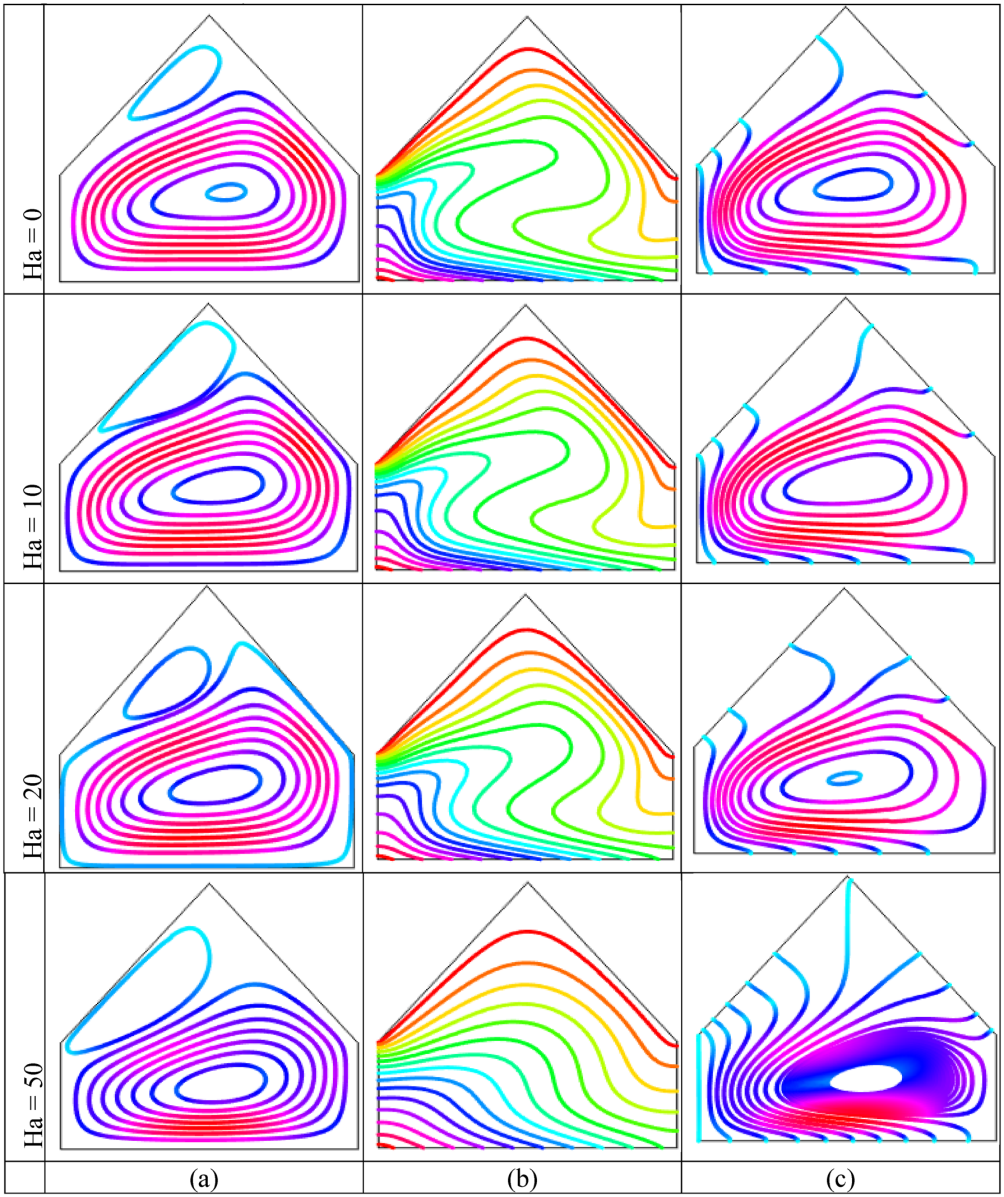
Outcomes of Hartmann number $(Ha)$ on (**a**) Streamline contours, (**b**) Isotherm contours (c) Heatlines for linear thermal boundary condition on bottom wall when $\phi = 0.04$ and $Ra = 10^{5}$.

The impact of Hartmann number (Ha) using streamlines, isotherms and heatlines are listed in Figs. 8 and 9 for uniform temperature condition and linear thermal boundary conditions, respectively when $Ra = 10^{5}$ and $\phi = 0.04$. This figures show that Hartmann number has significant impact on the field of temperature. Figure 8(a) shows the streamlines for different Hartmann number. This figures show a similar symmetric pattern of the streamlines for all considered values of Hartmann numer. The two symmetrical rotating cells are formed within the cavity where left cell rotates in anti-clockwise direction and right cells is rotating in clock-wise direction. The rotating strength decreases with the increase of Hartmann number i.e. the flow strength reduces with strong magnetic field. By applying an external magnetic field, a stronger field works over moving fluid which has a magnetic impressionability that weaken the flow circulation inside the enclosure. In addition, the Lorentz force generated by applying magnetic field that has a nature to oppose the varying its generation in case of the movement of the liquid and consequently, this force field weakens the streams inside the enclosure. Figure 8(b) shows that the isothermal lines are more and more distorted inside the cavity and denser near the bottom heated wall with the absence of Hartmann number. At low Hartmann number $(Ha = 0)$, higher temperature gradient is observed near bottom warmed wall. The denseness of the streamlines reduces with the influence of stronger magnetic field. Moreover, the isothermal lines moves upward more near the middle part of the bottom heated wall which indicates higher heat transport region.

A large circulation cell with a small tube in top corned of the enclosure is noticed in Fig. 9(a) for linear temperature condition. This rotating cell become smaller with rising influence of Hartmann number that indicates the decoction velocity by virtue of the effect of the Hartmann number. This happens due to the effect of magnetic field which retards the flow of the liquid. Figure 8(c) shows the heat transport characteristics of heatline method for the impact of Hartmann number. These figures show the flowing of heatline from bottom warmed wall to top cold walls. Two symmetrical rotating cells is observed for the absence of Hartmann number and lower Hartmann number. The heatlines are denser near the central vertical line of the cavity. The heat transport decreases with the increases of the intensity of Hartmann number. For Higher Hartmann number $(Ha = 50)$, the heatlines are flowing to top inclined walls from bottom heated wall. The flow heat is diminished because of the lower velocity for the higher intensity of the magnetic field. The similar pattern of the heatlines with a large central circular is observed for linear thermal condition in Fig. 9(c) for all value of Hartmann number.

**Figure 10:**
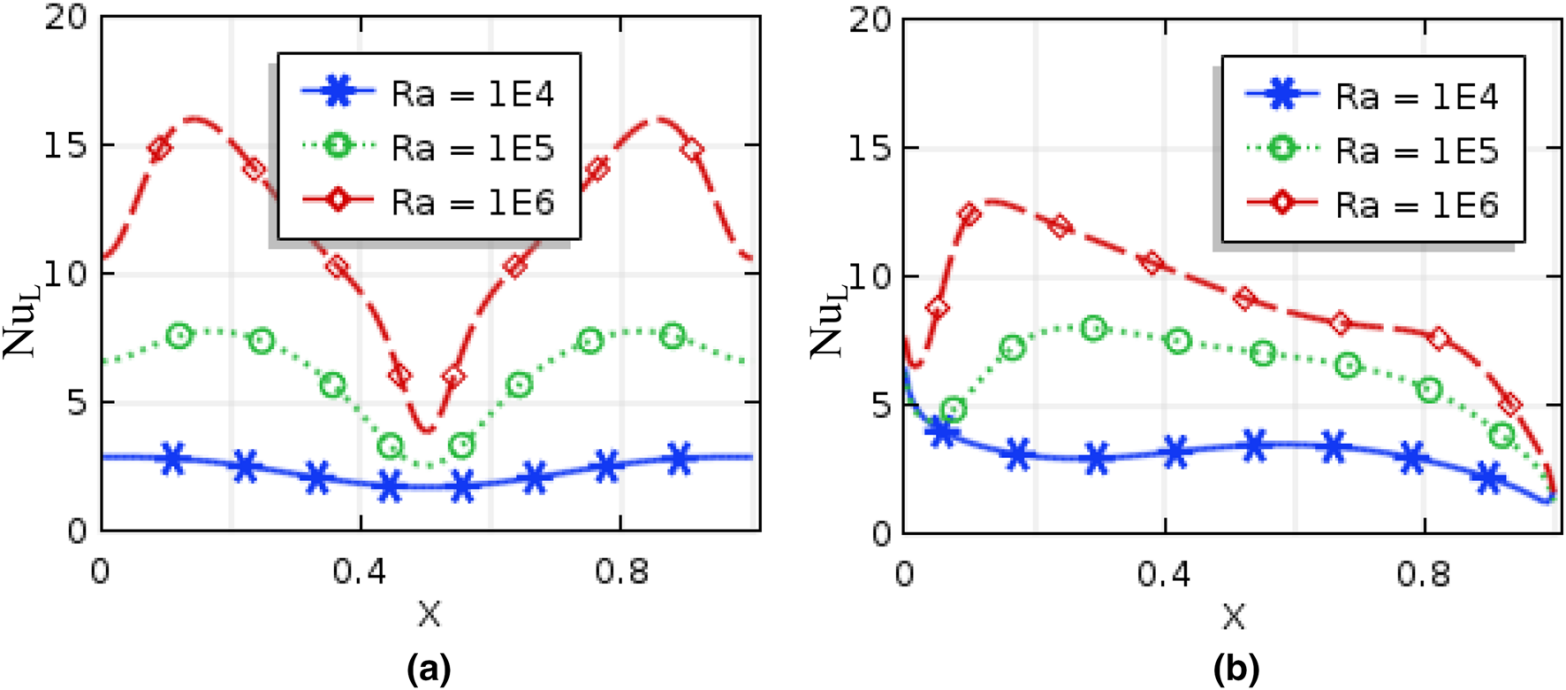
Local Nusselt number of Rayleigh number for (**a**) uniformly bottom heated wall (**b**) linearly heat bottom wall when $\phi = 0.04$ and $Ha = 10$.

**Figure 11:**
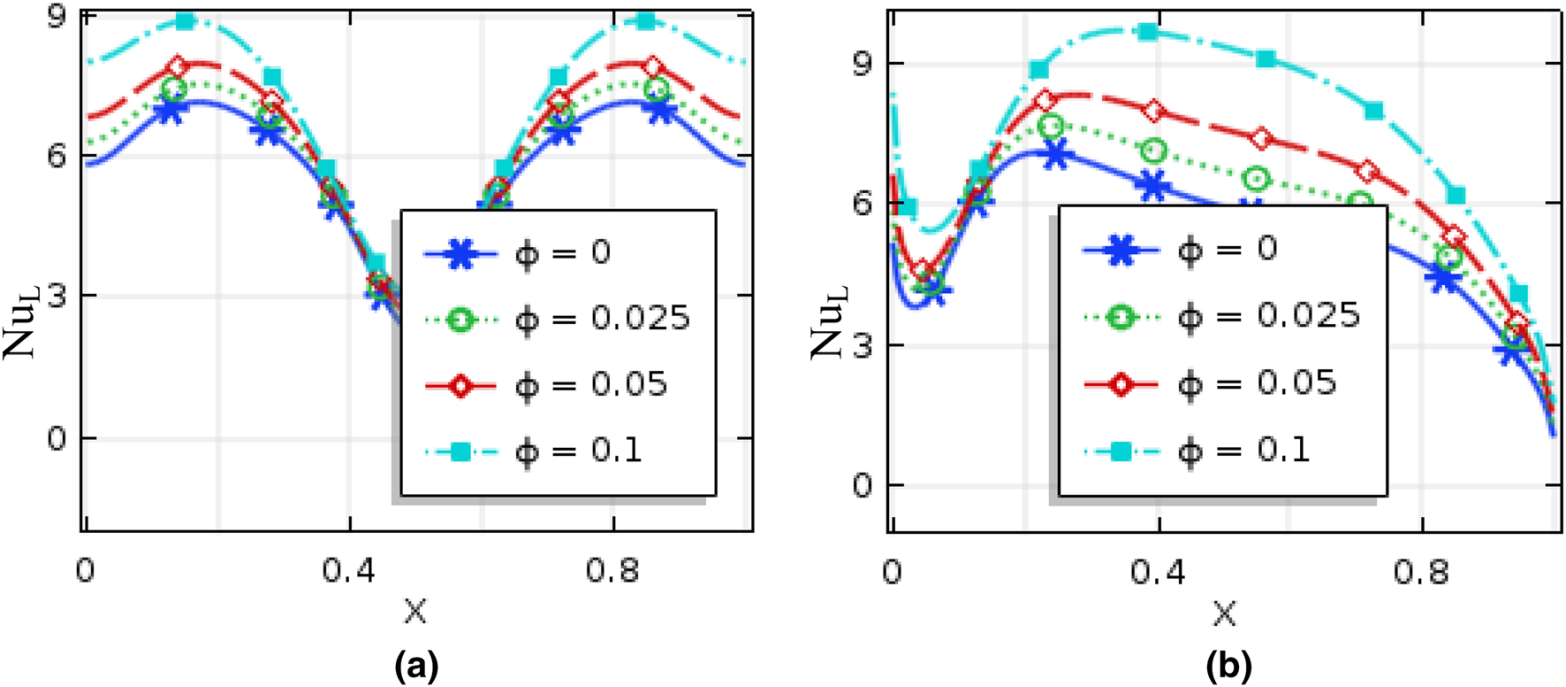
Local Nusselt number of nanoparticles volume fraction for (**a**) uniformly bottom heated wall (**b**) linearly heat bottom wall when $Ra = 10^{5}$ and $Ha = 10$.

**Figure 12:**
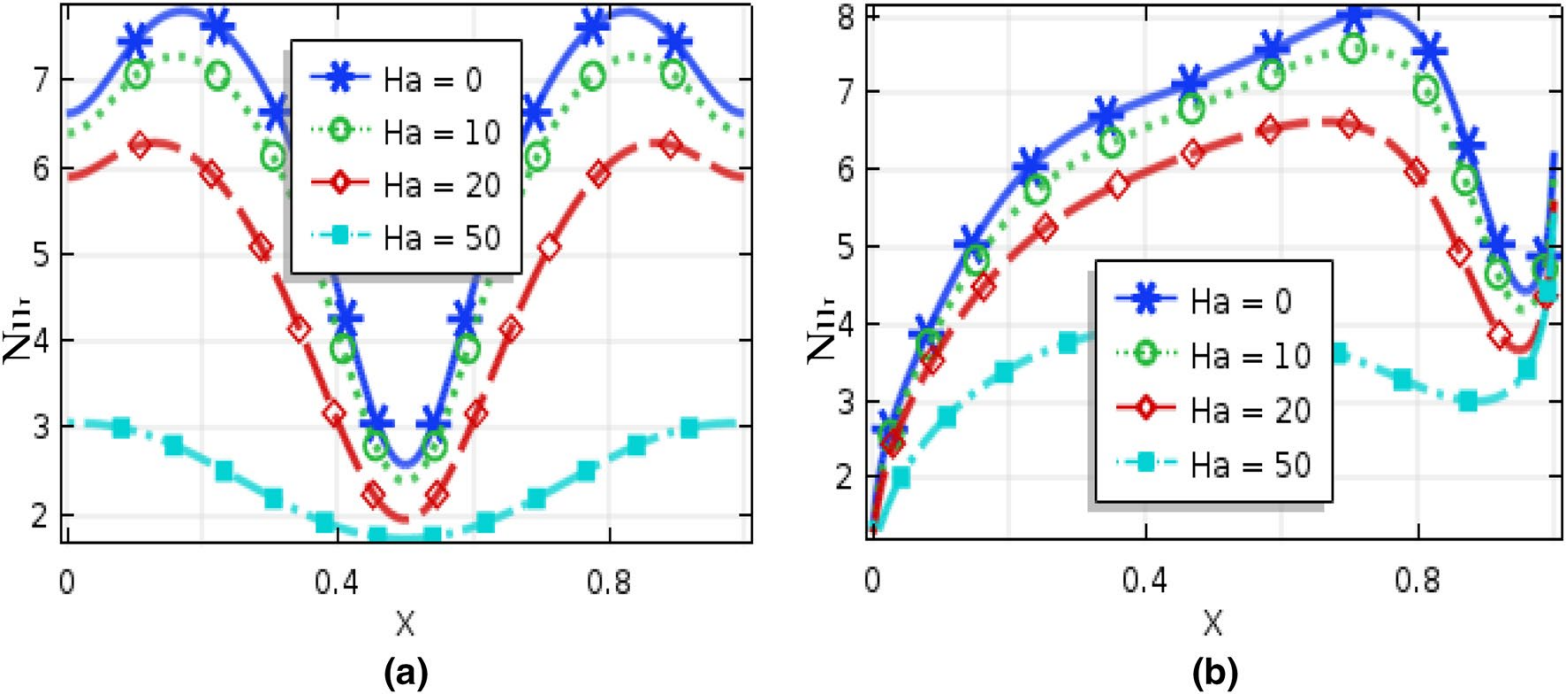
Local Nusselt number of nanoparticles volume fraction for (**a**) uniformly bottom heated wall (**b**) linearly heat bottom wall when $Ra = 10^{5}$ and $\phi = 0.04$.

Figures 10 and 11, respectively, represent local Nusselt number $(Nu_{L} )$ effects of $Ra$ and nanoparticles volume fractions on bottom hot wall for uniformly thermal boundary condition and linear temperature boundary condition. These figures narrate average rate of temperature transference enhances for the increment of both Rayleigh number and nanoparticles volume fractions for both uniformly thermal boundary condition as well as linearly thermal boundary condition. For low $Ra\,( = 10^{3} ,\,\,10^{4} )$, $(Nu_{L} )$ remain almost constant while the local Nusselt number increase for the dominant natural convection region $(Ra > 10^{4} )$ inside the enclosure. Figure 12 represent the local Nusselt number distributions along warmed wall for Hartmann number for both uniform and linear temperature boundary conditions. This figures show that the local Nusselt number diminishes rapidly for higher Hartmann number for uniform temperature system than linear temperature system. In addition, by changing the value of nanoparticles volume fraction, the Local Nusselt number is significantly affected.

**Figure 13:**
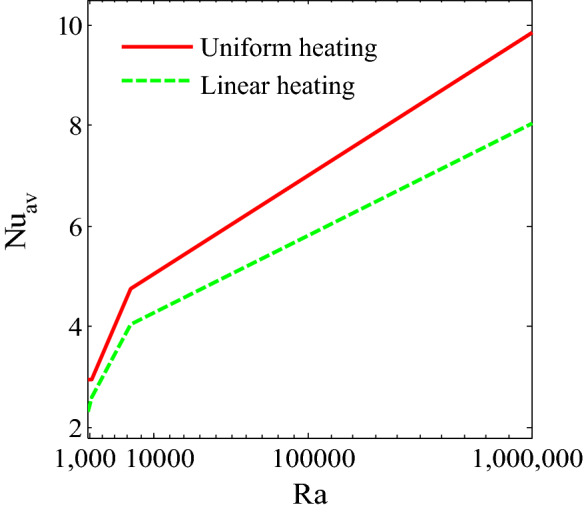
Average Nusselt number of Rayleigh number for two different heated boundary conditions at bottom wall.

**Figure 14:**
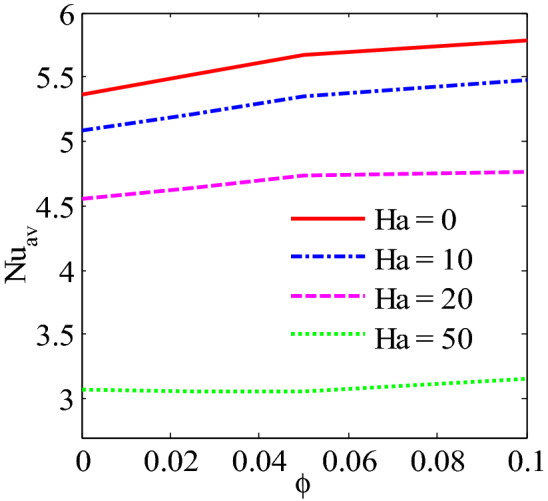
Average Nusselt number of nanoparticles volume fraction and Hartman number for Cu-H_2_O nanofluid.

**Figure 15:**
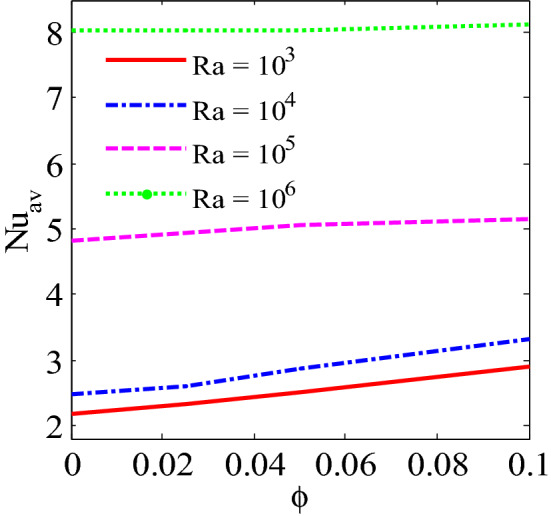
Average Nusselt number of nanoparticles volume fraction and Rayleigh number for Cu-H_2_O nanofluid.

**Figure 16:**
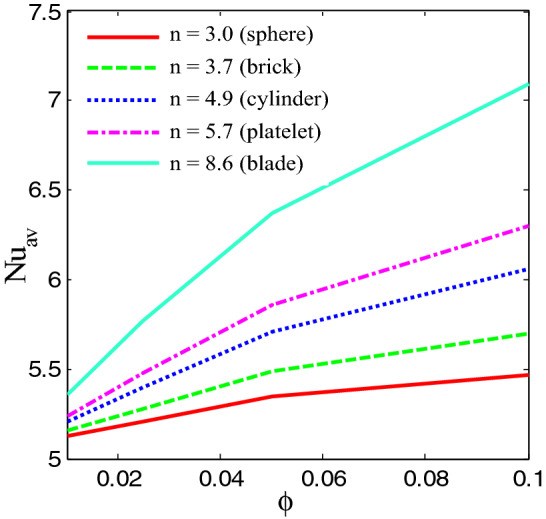
Average Nusselt number of nanoparticles volume fraction and different shape of nanaparticles for Cu-H_2_O nanofluid.

Figure 13 shows the average Nusselt number enhances monotonically by higher $Ra$. It is interesting observed that uniform heated condition on bottom wall provides highest temperature flow within the enclosure than linear temperature condition. Figures 14, 15 and 16 narrate that average Nusselt number increases with increases of Rayleigh number and addition of nanoparticles into the base fluid whereas decreases with the increase of Hartmann number. The rate of heat transport increase significantly with the increases of nanoparticles volume fraction. This is due to the higher thermal conductivity of nanofluids than base fluid. In addition, at low Ra, the influence of volume fraction of nanoparticles is more pronounced on heat transfer. Figure 16 depicts that rate of heat transfer is significantly higher for blade shape of nanoparticles than spherical shape of nanoparticles. This is due to the fact that less sphericity of the blade shape of nanoparticles. Moreover, the average Nusselt number is more apparent for higher nanoparticles volume fraction. Moreover, the average Nusselt number is higher for balde shape of nanoparticles compared to other all shape of nanoparticles such as spherical, brick, cylinder and platelet.

The parameter nanoparticles volume fraction is the key factor of the studying how nanoparticles affect the fluid flow as well as temperature transport of nanofluids. The types of nanoparticles is also a key factor for the enhancement of heat transfer. Table 3 illustrates average Nusselt number (Nu_av_) along bottom heated wall of the cavity for different value of nanoparticles volume fractions and different types of nanoparticles of base fluids such as water (H_2_O), kerosene and ethylene glycol (EG) with four different types of nanoparticles such as Cu, Co, Al_2_O_3_, and TiO_2_ when Ra = 10^5^, Ha = 15, and n = 3. It is observed that the average Nusselt number increases with the increases of nanoparticles volume fraction for all types of nanofluids. This is due to the higher thermal conductivity of nanoparticles. The Table depicts that highest heat transfer for kerosene based nanofluids compared to water-based and ethylene glycol based nanofluids. Although, Cu nanoparticles has higher thermal conductivity than Co, Al_2_O_3_, and TiO_2_, Co-kerosene nanofluids show higher rate of heat transfer. The lowest heat transfer is observed for water based nanofluids due to the lower thermal conductivity and higher dynamical viscosity of the based fluid.

**Table 3:** Average Nusselt number along bottom heated wall for different types of nanofluids and different size of the nanoparticles when $Ra = 10^{5}$ and $Ha = 10$.

Nanofluids	ϕ	Nu_av_	Increase (%)	Nanofluids	ϕ	Nu_av_	Increase (%)
Cu-water	0.00	5.0869043	–	Cu-kerosene	0.00	5.1108449	–
	0.01	5.1279040	0.81		0.01	5.3390452	4.46
	0.05	5.3482914	5.14		0.05	5.5897890	9.37
	0.10	5.4715713	7.56		0.10	5.7623863	12.75
Co-water	0.00	5.0869043	–	Co-kerosene	0.00	5.1108449	–
	0.01	5.1255934	0.76		0.01	5.4048901	5.75
	0.05	5.3361810	4.90		0.05	5.7791370	13.08
	0.10	5.4451034	7.04		0.10	5.9494259	16.41
Al_2_O_3_-water	0.00	5.0869043	–	Al_2_O_3_-kerosene	0.00	5.1108449	–
	0.01	5.1493271	1.23		0.01	5.2676440	3.07
	0.05	5.4261935	6.67		0.05	5.5797743	9.18
	0.10	5.7008267	12.07		0.10	5.8481775	14.43
TiO_2_-water	0.00	5.0869043	–	TiO_2_-kerosene	0.00	5.1108449	–
	0.01	5.1394618	1.04		0.01	5.1601554	0.96
	0.05	5.3977902	6.11		0.05	5.4457005	6.55
	0.10	5.6101714	10.29		0.10	5.7818382	13.13
Cu-EG	0.00	5.1015913	–	Al_2_O_3_-EG	0.00	5.1015913	–
	0.01	5.1390452	0.73		0.01	5.1662068	1.27
	0.05	5.3897890	5.649		0.05	5.4683156	7.19
	0.10	5.5339510	8.47		0.10	5.8291424	14.27
Co-EG	0.00	5.1015913	–	TiO_2_-EG	0.00	5.1015913	–
	0.01	5.1391603	0.74		0.01	5.1564652	1.08
	0.05	5.3895143	5.64		0.05	5.4205563	6.25
	0.10	5.5662212	9.18		0.10	5.7300720	12.32

## Conclusions

The convective flow and temperature into cupper-water nanofluid within a prismatic enclosure to visualize the heatline has been investigated considering two various heated boundary conditions. The numerical methods finite element analysis of Galerkin weighted residual form is executed. The outcomes for model parameters on flow field as well as temperature flow are presented using streamline contours, isothermal contours, heatlines, local and average Nusselt numbers and interpreted. The main outcomes are listed below:

- At low Rayleigh number, the elementary form of temperature flow is conduction and influence nanoparticles are more pronounced on heat transfer.
- Convective temperature flow enhances significantly for the augmentation of Rayleigh number.
- Flow field are strongly affected within the cavity for higher Rayleigh number.
- Better temperature flow is performed for upper Rayleigh number through convection.
- Addition nanoparticles into base fluid increases heat transfer rate.
- The average temperature transport rate increases with additional nanoparticles volume fraction.
- The uniform mode of thermal boundary condition represents the highest temperature transfer rate for copper–water nanofluid.
- The increasing intensity of Hartmann number reduces fluid velocity as well as heat flow.
- Effective heat transport can be obtained with the existence of nanoparticles. The kerosene based nanoparticles shows highest heat transfer rate compared to other nanofluids.
- The shape of nanoparticles is a key factor of heat transport. Higher rate of heat transport is observed for blade shape of nanoparticles.

## List of symbols

*B*_0_: Magnetic field strength
*c*_*p*_: Specific heat at constant pressure
*g*: Acceleration of gravitation
*Ha*: Hartmann number
*k*: Thermal conductivity
*L*: Cavity length
*Nu*_*av*_: Average Nusselt number
*Nu*_*L*_: Local Nusselt number
*p*: Dimensional pressure
*P*: Non-dimensional pressure
*Pr*: Prandtl number
*Ra*: Rayleigh number
*T*: Fluid temperature
*u, v*: Dimensional velocity component
*U, V*: Dimensionless velocity component
*x, y*: Dimensional coordinates
*X, Y*: Non-dimensional coordinates

## Greek symbols

*α*: Thermal diffusivity
*β*: Thermal expansion coefficient
*ψ*: Stream function
*ϕ*: Solid volume fraction
*μ*: Dynamic viscosity
*υ*: Kinematic viscosity
*θ*: Non-dimensional temperature
*ρ*: Density
*σ*: Electric conductivity

## Subscript

*h*: Heat surface
*c*: Cold surface
*nf*: Nanofluid
*bf*: Basefluid
*sp*: Solid particle
*L*: Local
